# The Role of Visual and Semantic Properties in the Emergence of Category-Specific Patterns of Neural Response in the Human Brain

**DOI:** 10.1523/ENEURO.0158-16.2016

**Published:** 2016-08-01

**Authors:** David D. Coggan, Daniel H. Baker, Timothy J. Andrews

**Affiliations:** Department of Psychology and York Neuroimaging Centre, University of York, York YO10 5DD, United Kingdom

**Keywords:** category, EEG, image, MVPA, object

## Abstract

Brain-imaging studies have found distinct spatial and temporal patterns of response to different object categories across the brain. However, the extent to which these categorical patterns of response reflect higher-level semantic or lower-level visual properties of the stimulus remains unclear. To address this question, we measured patterns of EEG response to intact and scrambled images in the human brain. Our rationale for using scrambled images is that they have many of the visual properties found in intact images, but do not convey any semantic information. Images from different object categories (bottle, face, house) were briefly presented (400 ms) in an event-related design. A multivariate pattern analysis revealed categorical patterns of response to intact images emerged ∼80–100 ms after stimulus onset and were still evident when the stimulus was no longer present (∼800 ms). Next, we measured the patterns of response to scrambled images. Categorical patterns of response to scrambled images also emerged ∼80–100 ms after stimulus onset. However, in contrast to the intact images, distinct patterns of response to scrambled images were mostly evident while the stimulus was present (∼400 ms). Moreover, scrambled images were able to account only for all the variance in the intact images at early stages of processing. This direct manipulation of visual and semantic content provides new insights into the temporal dynamics of object perception and the extent to which different stages of processing are dependent on lower-level or higher-level properties of the image.

## Significance Statement

Previous studies have shown distinct spatial and temporal patterns of response to different object categories. However, the extent to which these patterns are based on lower-level visual properties compared with high-level semantic information remains unclear. To address this question, we used scrambled objects that preserve visual properties but do not convey any semantic information. We found distinct patterns of response to intact images from different object categories. Patterns of response to scrambled images from different categories emerge in a way that is similar to those of intact images but do not persist for the same duration. These findings demonstrate the relative importance of both lower-level visual and higher-level semantic properties in the neural response to objects.

## Introduction

A variety of evidence has shown that spatially distinct regions of the ventral occipitotemporal cortex are selective for different categories of objects (Kanwisher, 2010). Lesions to this region often result in difficulties in recognizing and naming specific object categories (Farah, 1990; McNeil and Warrington, 1993; Moscovitch et al., 1997). The notion that discrete areas of the temporal lobe are specialized for different categories of objects receives support from functional imaging studies that show that some regions in the temporal lobe are more responsive to faces than to other categories (Allison et al., 1994; Kanwisher et al., 1997). Other imaging studies have found similar category-specific responses for inanimate objects (Malach et al., 1995), scenes (Epstein and Kanwisher, 1998), and human body parts (Downing et al., 2001). Although specialized regions have only been reported for a limited number of objects, the spatial pattern of response across the entire ventral stream can distinguish a wider range of categories (Haxby et al., 2001; Kriegeskorte et al., 2008; Connolly et al., 2012; Clarke and Tyler, 2014; Rice et al., 2014).

A full understanding of object perception requires the ability to discriminate object-specific brain states with both spatial and temporal resolution. Recently, reliable patterns of neural response to images from different object categories have been shown with MEG and EEG (Carlson et al., 2011, 2013; Cauchoix et al., 2014; Cichy et al., 2014; Clarke et al., 2015). These techniques complement previous MRI studies by providing temporal information about when these categorical patterns of response emerge and how long they are sustained. Temporal properties are important, as they place constraints on models of object recognition (Mur and Kriegeskorte, 2014). Such models suggest a dynamic process in which there is a transformation from a visual representation (based on the statistics of the image) to a semantic representation (reflecting the meaning of the object; Clarke and Tyler, 2014). It is thought that the initial component of the response reflects fast feedforward processing that is related to visual properties, whereas later patterns reflect recurrent processing that might be related to semantic properties of the stimulus (Lamme and Roelfsema, 2000; Hochstein and Ahissar, 2002; Bar et al., 2006; DiCarlo and Cox, 2007).

The aim of this study was to investigate the relative importance of visual and semantic properties of objects in the emergence of categorical patterns of neural response. However, a fundamental problem in this endeavor is that the visual and semantic properties of objects often covary, making it difficult to resolve the relative contribution of these sources of information to patterns of neural response. So, it is not clear from many previous studies whether the distinct patterns of response to different object categories reflect visual or semantic properties (Carlson et al., 2011, 2013; Cauchoix et al., 2014; Cichy et al., 2014). In a recent MEG study, Clarke et al. (2015) addressed this issue by showing that the categorization of objects based on the neural response could be predicted by the visual properties of the image. However, they also found that accuracy could enhanced by including semantic properties, particularly at later stages of processing. Although this suggests that visual and semantic properties are both important for the neural representation of objects, this approach is not able to show a causal link.

To address this issue, we measured patterns of EEG response to intact images from different object categories, as well as versions of these images that had been phase scrambled on a global or local basis. Our rationale for using scrambled images is that they have many of the visual properties found in intact images, but they do not convey any semantic information (Coggan et al., 2016). This allows us to determine the extent to which the preserved visual properties contribute to the neural representation of objects in the absence of any semantic content. The comparison between the locally scrambled and globally scrambled images also allows us to explore the importance of spatial image properties, which are preserved in the locally scrambled condition. In a recent fMRI study, we found similar spatial patterns of response to intact and scrambled images across the ventral visual pathway (Coggan et al., 2016). This study demonstrated the importance of low-level visual properties in the patterns of response in the ventral visual pathway. By comparing the similarity of the responses to intact and scrambled images using EEG, we aim to determine the relative contribution of visual properties to categorical patterns of response at different time points.

## Materials and Methods

### Stimuli

**Figure 1.**
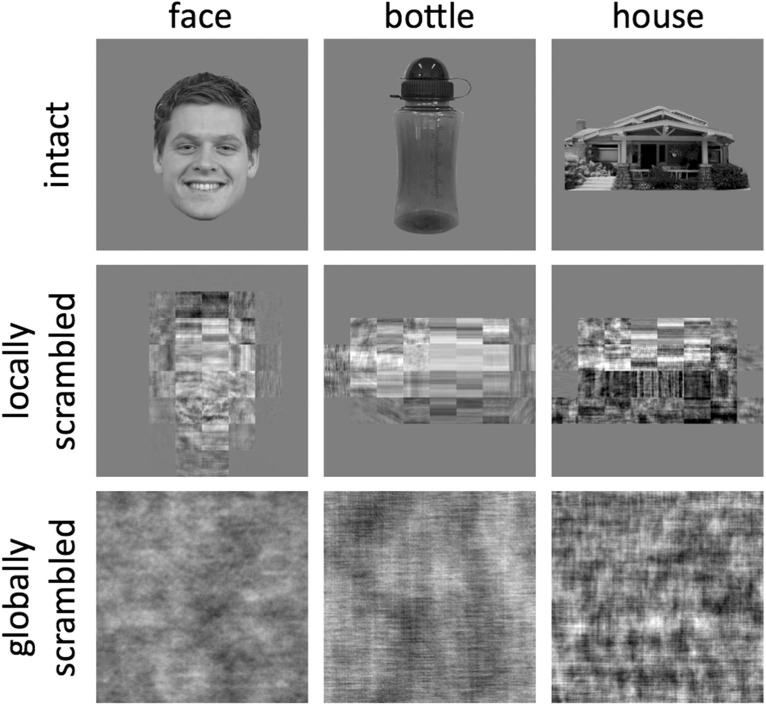
Exemplars of intact, locally scrambled, and globally scrambled images from the different object categories.

On hundred five images of three object categories (face, bottle, and house) were taken from an object–image stimulus set (Rice et al., 2014). All images were grayscale, were superimposed on a middle gray background, and had a resolution of 400 × 400 pixels (Fig. 1). For each of these original images, two different phase-scrambled versions were generated. A global-scrambling method involved a typical Fourier scramble (i.e., keeping the global power of each two-dimensional frequency component constant while randomizing the phase of the components). A local-scrambling method involved windowing the original image into an 8 × 8 grid and applying the phase scramble to each 50 × 50 pixel window independently. In a previous study (Coggan et al., 2016), we showed that these scrambling methods effectively remove any semantic or categorical content in the images. Stimuli were presented using a gamma-corrected VIEWPixx display (VPixx Technologies) with a resolution of 1920 × 1200 pixels and a refresh rate of 120 Hz. Images were viewed at a distance of ∼57 cm and subtended a retinal angle of 8°.

### Participants

Twenty participants (3 males; mean age, 20.6 years; SD, 2.6 years) with normal or corrected-to-normal vision took part in the experiment. Participants gave written, informed consent. The study was approved by the University of York Department of Psychology Ethics Committee. The data for one participant (female) were removed from the analysis due to partial data loss.

### Design and procedure

The experiment involved the following three runs: the first run contained globally scrambled images; the second run contained locally scrambled images; and the third run contained intact images. Therefore, participants were unaware of the object categories in our stimulus set prior to viewing the scrambled images. Each run contained 35 blocks. There were 10 trials in each block. In each trial, an image from one of the three object categories was presented for 400 ms. There was a jittered intertrial interval that had a mean duration of 1 s and an SD of 200 ms. The duration of the interblock interval was 3 s. Participants fixated a cross in the center of the screen between trials. To maintain attention, participants were instructed to click a mouse whenever a red dot appeared on an image. One image in each block contained a red dot. Self-timed rests were taken between runs.

### EEG recording

EEG waveforms were recorded from 64 scalp locations laid out according to the 10/20 system in a WaveGuard cap (ANT Neuro). Data from each electrode were referenced against a whole-head average. We also monitored blinks through bipolar electro-oculogram (EOG) electrodes placed above and below the left eye. Signals were amplified and digitized at 1000 Hz and recorded using the ANT Neuroscan software (ANT Neuro). Stimulus-contingent triggers were sent from the VIEWPixx device to the EEG amplifier using a 25-pin parallel port with microsecond-accurate synchronization to the display refresh sequence. The PsychToolbox routines (Brainard, 1997; Pelli, 1997) running in Matlab were used to control the display hardware and to send triggers.

### EEG preprocessing

The EEG traces from each run were concatenated and bandpass filtered between 0.01 and 30 Hz prior to epoching. Blink artifacts were corrected using independent components analysis (ICA). This involved running ICA across data from all electrodes, including the vertical EOG, and manually selecting the components that captured blink artifacts. These components were then subtracted from the EEG trace at each electrode site according to their weighting. This approach meant that no trials were rejected. The EEG trace was then divided into epochs ranging from 200 ms before stimulus onset to 800 ms after stimulus onset. All trials containing a red dot were removed prior to further analysis.

### EEG MVPA analysis

All data processing was performed in Matlab using custom scripts. To measure the spatial patterns of EEG response for each participant, trials were collapsed into mean ERPs for odd and even trials for each condition and at each electrode site. These condition-averaged ERPs were then baselined by subtracting the mean amplitude during the 200 ms prior to stimulus onset (across both odd and even trials) from the response at each time point. From these ERPs, a 64-value vector representing the spatial pattern of response across all electrodes was extracted for odd and even trials for each object category at each time point.

Pattern vectors were normalized within each participant using the following method. First, vectors were selected from one time point and one image type. This gave a total of six patterns (odd/even × face/bottle/house). For each electrode site, the mean amplitude across all six patterns was subtracted from its amplitude in each pattern. This process was repeated for each image type at each time point.

**Figure 2.**
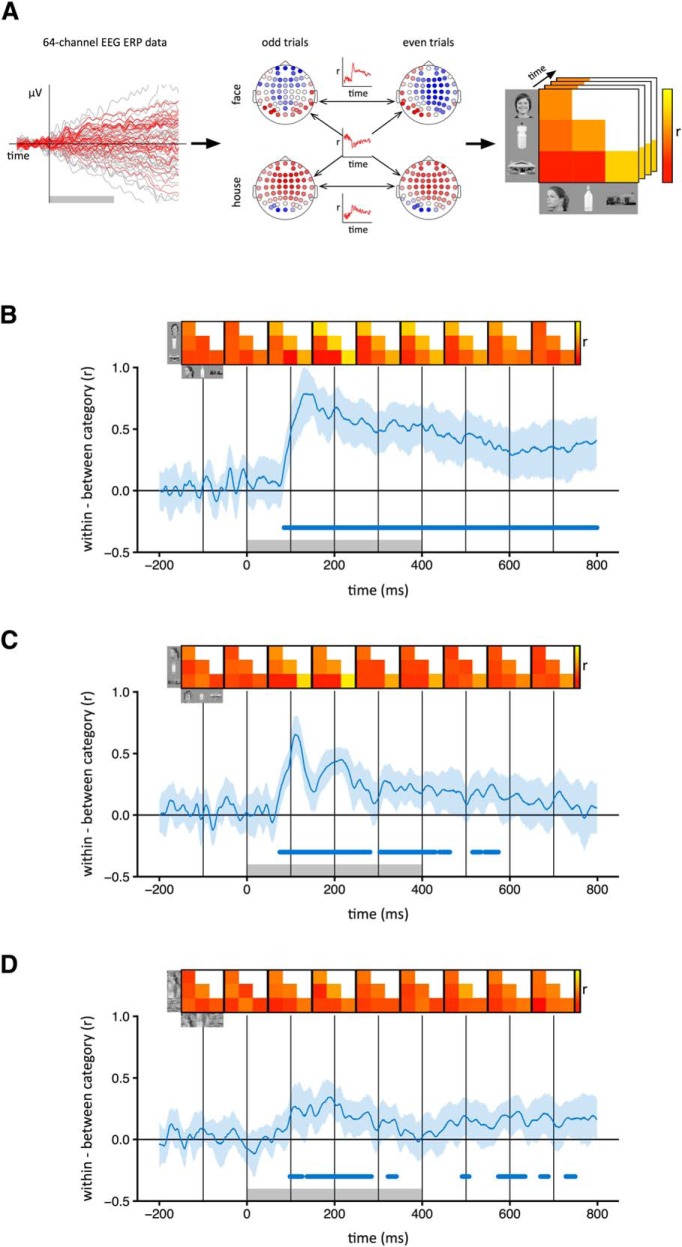
Category-specific patterns of EEG response to intact and scrambled images. ***A***, For each time point, normalized patterns of response to odd and even trials of each category were compared across 64 electrodes. The correlation coefficients were then represented in a similarity matrix for that time point. Distinct category-specific patterns of response were defined by higher within-category (e.g., face–face) compared to between-category (e.g., face–bottle) correlations. ***B–D***, Correlation time-courses are shown for the intact (***B***), locally scrambled (***C***), and globally scrambled (***D***) image types. The shaded region represents 95% CIs obtained by bootstrapping across participants. Group mean correlation matrices at 100 ms intervals are shown above the plot. Gray box at the base of the plot represents the time points at which the stimulus was present. Blue bar at the base of the plot represents time points at which the lower bound of the CI is >0, indicating significantly higher within-category correlations than between-category correlations.

To see whether different object categories evoke distinct patterns of EEG response, we ran a correlation-based MVPA separately for each image type and time point (Fig. 2*A*). This involved measuring the correlation between pattern vectors within and among the three object categories. For within-category correlations (e.g., face vs face), we measured the correlation between odd and even trials. For between-category correlations (e.g., bottle vs house), we used the mean correlation between odd trials of the first category and even trials of the second, and between even trials of the first category and odd trials of the second. The distinctiveness of the patterns of EEG response was then measured by subtracting between-category correlations from within-category correlations. The 95% confidence intervals (CIs) for this difference were then obtained by bootstrapping across participants. Points at which different object categories evoked significantly distinct patterns of EEG response were defined by the lower confidence interval being >0.

To measure the similarity between responses to intact and scrambled images from the same object category, we first collapsed patterns across odd and even trials to create one pattern per condition per time point. We then correlated the patterns of response at each time point separately for the intact locally scrambled and the intact globally scrambled contrasts for each category. A group mean was calculated across categories, and 95% confidence intervals were obtained by bootstrapping across participants.

To determine whether the response to intact images could be explained by the response to scrambled images, we calculated a noise ceiling. This estimates that maximum correlation that could be expected. The noise ceiling was calculated by measuring the correlation between the responses at odd and even trials within each category in the intact condition. At the individual level, we take a mean of the within-category correlations (face–face, bottle–bottle, house–house) for each time point. We then average across subjects to obtain one noise ceiling estimate at each time point. Time points at which this value fell within the 95% CI for the correlation between intact and scrambled images demonstrate when all the variance in the intact images was explained by the scrambled images.

The correlation-based method was complemented with a classification-based approach involving a support vector machine, producing similar results. To see whether different object categories evoked distinct patterns of response, classification was performed separately for each participant, image type, and time point (see Fig. 6). First, patterns of EEG response were extracted for each trial of each category. Two “training” patterns and one “testing” pattern for each category were generated by randomly dividing the 105 trials into three equal sets and taking an average. A support vector machine was then trained on the six training patterns, and tested on the three testing patterns. This procedure was repeated 100 times, with different subsets of trials used for training and testing in each iteration. To see whether similar patterns of response were evoked by intact and scrambled images from the same category, the classifier was altered so that test patterns were substituted with those from another image type. This was performed for each pairwise contrast between image types, and accuracy was averaged across both directions (e.g., train on intact, test on locally scrambled; and train on locally scrambled, test on intact).

Finally, to examine transient and persistent neural activity in response to each condition, we conducted a temporal cross-correlation. This involved measuring the correlation between response patterns for odd and even trials for the same condition, iterating over each possible pair of time points. Correlations were represented in a 1000 × 1000 similarity matrix, and data were averaged across the positive diagonal. Matrices were then collapsed across categories to give one matrix per image type.

## Results

**Figure 3.**
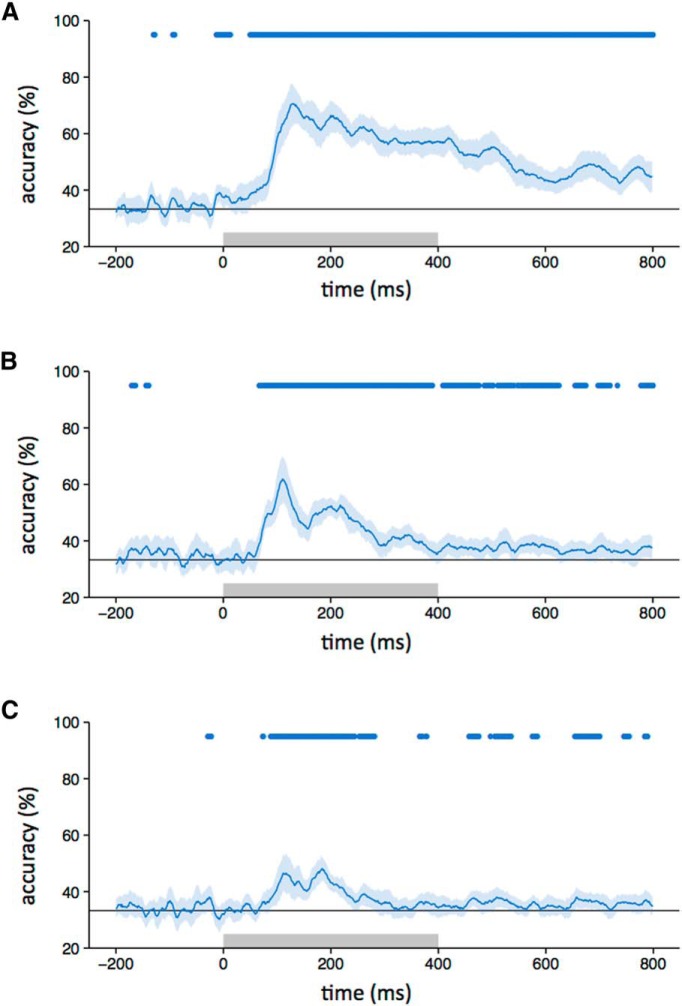
***A–C***, Classifier accuracy for between-category discrimination (blue line) with intact (***A***), locally scrambled (***B***), and globally scrambled (***C***) images (chance = 33%, gray line). The blue-shaded regions represent 95% CIs obtained through bootstrapping across participants. The blue bar at the top of the plot represents time points at which the lower bound of the CI is above chance. The gray box on the axes of the plot represents the stimulus duration.

First, we asked whether different intact object categories produced distinct spatial patterns of EEG response (Fig. 2). To address this question, we compared the similarity of patterns of response to images from the same category (e.g., face vs face) with the similarity of patterns to images of different categories (e.g., face vs house). Categorical patterns of response were demonstrated when the within-category correlations were significantly greater than the between-category correlations. Categorical patterns of response to intact images emerged 80 ms after stimulus onset. The patterns were maximally distinct at ∼150 ms and persisted until at least 800 ms (Fig. 2*B*). A classification-based approach was then used to complement the correlation-based method. In this analysis, a classifier was trained on a subset of the data and tested on the remaining data. This showed a pattern that was similar to that derived from the correlation-based analysis. Accuracy above the level of chance emerged 80 ms after stimulus onset, peaked at about 150 ms, and persisted until 800 ms (Fig. 3*A*).

To measure the extent to which these category-specific patterns of response were based on lower-level visual properties, we first asked whether locally scrambled and globally scrambled images also produced distinct category-specific patterns of EEG response using both the correlation-based (Fig. 2*C*,*D*) and classification-based (Fig. 3*B*,*C*) analyses. Distinct category-specific patterns of response for locally scrambled images emerged at ∼80 ms after stimulus onset. They were maximally distinct at ∼110 ms and persisted until ∼400–500 ms. Distinct category-specific patterns of response for globally scrambled images emerged at ∼100 ms after stimulus onset. They were maximally distinct at ∼190 ms and persisted until ∼300 ms.

**Figure 4.**
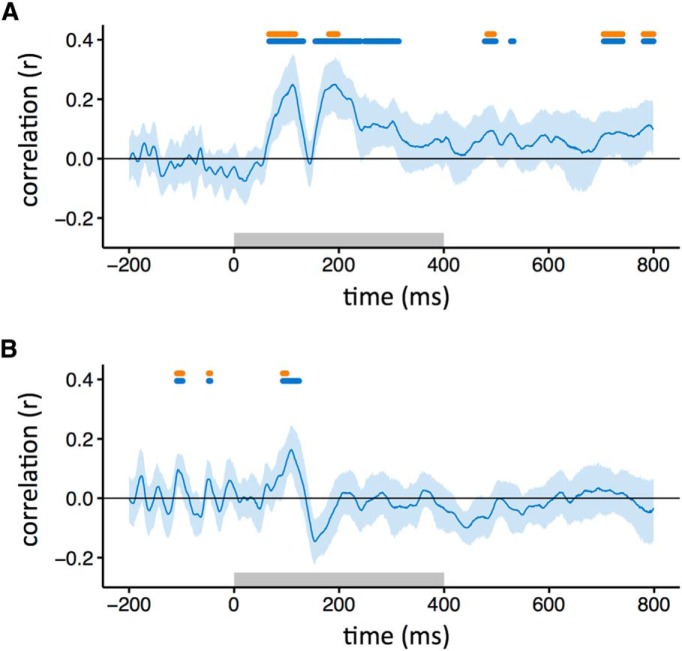
Similarity between patterns of EEG response to intact images and locally scrambled (***A***) or globally scrambled (***B***) images from the same object category. Blue-shaded regions represent 95% CIs across participants. The blue bar at the top of the plot indicates time points at which the correlation is significantly >0. The orange bar indicates the time points at which the correlation is not significantly different from the noise ceiling. The gray box represents the stimulus duration.

**Figure 5.**
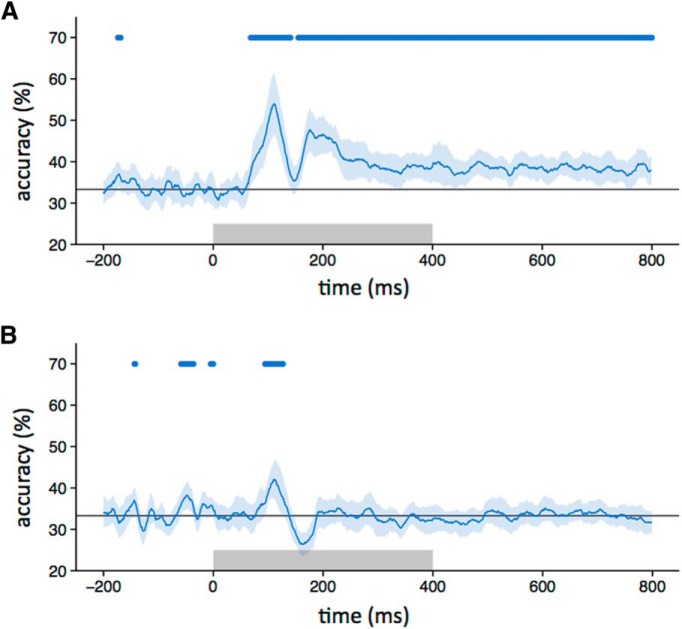
Classifier performance across different image types. ***A***, Accuracy in classifying responses to either intact or locally scrambled images when trained on locally scrambled or intact images, respectively. ***B***, Accuracy in classifying responses to either intact or globally scrambled images when trained on globally scrambled or intact images, respectively. The blue line indicates classifier accuracy across time, with shaded regions representing 95% CIs obtained through bootstrapping across participants. The blue bar at the top of the plot represents the time points at which the lower bound of the CI is above chance. The gray box shows stimulus duration.

Although distinct patterns of response were evident to scrambled images from different categories (i.e., within-category > between-category correlations), it is not clear whether the patterns were similar to those elicited from the intact images. To address this question, we correlated patterns of response to the same object category across different levels of scrambling at different time points. Figure 4*A* (blue horizontal bar) shows that the correlation between intact and locally scrambled images became significant at ∼80 ms after stimulus onset, and peaked at ∼110 and 190 ms. The percentage duration that the locally scrambled patterns were correlated with the intact patterns was greater during the stimulus period (0–400 ms, 27%) compared with the poststimulus period (400–800 ms, 10%). A similar pattern of results was evident when we trained a classifier on intact or locally scrambled images and then tested on locally scrambled or intact images, respectively (Fig. 5*A*). The duration of accuracy above the level of chance with the locally scrambled and intact conditions was similar during the stimulus period (0–400 ms, 40%) and the poststimulus period (400–800 ms, 49%).

Next, we explored the similarity between the intact and globally scrambled images (Figs. 4*B*, 5*B* ). The correlation between responses to intact and globally scrambled images became significant (blue horizontal bar) ∼90 ms after stimulus onset, peaked at ∼110 ms, and persisted until ∼120 ms. The percentage duration that the locally scrambled patterns were correlated with the intact patterns was greater during the stimulus period (0–400 ms, 4%) compared with the poststimulus period (400–800 ms, 0%). A similar pattern of results was evident when we trained a classifier on intact or locally scrambled images and then tested it on locally scrambled or intact images, respectively (Fig. 5*A*). The duration of accuracy above the level of chance with the locally scrambled and intact conditions was greater during the stimulus period (0–400 ms, 4%) compared with the poststimulus period (400–800 ms, 0%).

To directly compare the similarity between intact images and either locally scrambled or globally scrambled images, the average correlation (Fig. 4) or accuracy (Fig. 5) was compared across individuals. The average correlation between intact and locally scrambled images was significantly higher than the correlation between intact and globally scrambled images (*t*_(18)_ = 3.29, *p* < 0.005). Similarly, the average accuracy (Fig. 5) with intact and locally scrambled images was significantly higher than with intact and globally scrambled images (*t*_(18)_ = 5.34, *p* < 0 .0001).

We then asked whether the explainable variance in intact responses was fully accounted for by the responses to scrambled images, given the level of noise in the data. This was achieved by calculating a noise ceiling (Nili et al., 2014). This involved measuring the correlation to intact images from the same category across odd and even trials of the same category. The noise ceiling was not fixed, but varied across time. We then determined whether the correlation between intact and scrambled images was not significantly different from the noise ceiling for each time point. For locally scrambled images, the 95% CIs of the correlations overlapped until ∼120 ms after stimulus onset (Fig. 4*A*). The percentage duration that the locally scrambled patterns were not significantly different from the noise ceiling was similar during the stimulus period (0–400 ms, 9%) and the poststimulus period (400–800 ms, 9%). For globally scrambled images, the confidence intervals overlapped until ∼100 ms after stimulus onset (Fig. 4*B*). The percentage duration that the globally scrambled patterns were not significantly different from the noise ceiling was greater during the stimulus period (0–400 ms, 1%) compared with the poststimulus period (400–800 ms, 0%).

**Figure 6.**
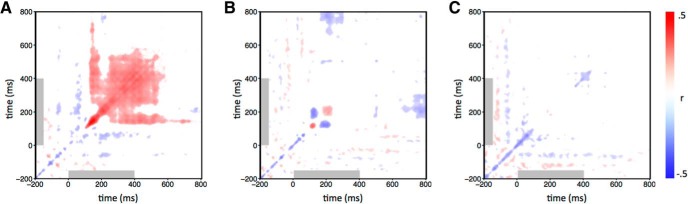
Temporal cross-correlation matrices for each image type. Responses to trials of the same condition were correlated over each combination of time points. ***A–C***, Correlations were collapsed across categories to give one matrix per image type [intact (***A***), locally scrambled (***B***), globally scrambled (***C***)]. The colorbar represents Pearson’s correlation coefficients. Matrices were thresholded by obtaining 95% CIs at each coordinate by bootstrapping across participants. Coordinates at which these intervals overlapped with 0 are shown in white. The gray box represents the stimulus duration.

Finally, we investigated the stability of the category-specific patterns of response for each image manipulation (Cichy et al., 2014). This involved measuring the correlation between the patterns of EEG response within each condition across different time points. The results were then averaged across categories for each image type and represented in time–time similarity matrices (Fig. 6). Here, the diagonal for intact images corresponds to the noise-ceiling estimate used in Figure 4. For intact images, the pattern of response from 100 to 150 ms was positively correlated with patterns found from ∼250 to 600 ms. The continuation of this neural activity far beyond stimulus offset suggests that this does not reflect prolonged visual input during image presentation. The locally scrambled matrix shows no evidence of persistent neural activity as seen in the intact matrix, but does exhibit transient neural activity between ∼100 and 250 ms after stimulus onset. Interestingly, time point combinations of ∼150 and ∼200 ms show negative correlations, suggesting a polarity reversal in the potentials between these latencies. The globally scrambled matrix shows weak correlations across all combinations of time points.

## Discussion

The aim of this study was to determine the contribution of lower-level visual and higher-level semantic properties to the emergence of categorical patterns of neural response. To address this question, we compared patterns of EEG response to intact and scrambled images from different object categories. Scrambled images were used because they contain visual properties similar to those of intact images but do not convey any semantic information (Coggan et al., 2016). Our results show similar category-specific patterns of response at early stages of processing. However, these patterns were sustained for a longer time with intact images compared with scrambled images. These results show the importance of visual properties in the emergence of categorical patterns of response, but also show the importance of semantic properties in the recurrent processing that sustains these patterns.

The emergence of category-specific patterns of EEG response to intact images is comparable to previous studies using MEG that found that categorical distinctions can be decoded prior to 100 ms after stimulus onset and become maximally distinct at ∼140 ms (Carlson et al., 2013; Cauchoix et al., 2014; Cichy et al., 2014). However, most previous studies have not directly determined whether these patterns of response reflect lower-level visual properties or higher-level semantic properties of the image. Recently, Clarke et al. (2015) addressed this issue with MEG showing that visual properties can explain patterns of response to different categories of objects. However, they also showed that the semantic properties of objects were able to explain additional variance in the pattern of response, particularly at later stages of the response. In our study, we were also able to show that the patterns of response to images from different object categories are driven predominantly by the lower-level visual properties at early stages of visual processing (up to 150 ms). Visual properties were also able to partially account for the variance in the response to intact images at later stages of processing.

Patterns of response to intact images were correlated more strongly and for a longer period of time with responses to locally scrambled images than with globally scrambled images. One key difference between these two conditions is that the spatial properties, such as the shape (or spatial envelope) of the image, are somewhat preserved in the locally scrambled images, but not in the globally scrambled images. In a recent fMRI study, we showed that the spatial pattern of response in the ventral stream to different categories of intact objects was more similar to the pattern elicited by locally scrambled objects compared with globally scrambled objects. The greater similarity between responses to intact locally scrambled images is consistent with previous studies that have shown a modulatory effect of spatial properties on patterns of response in the ventral visual pathway (Levy et al., 2001; Golomb and Kanwisher, 2012; Silson et al., 2015; Bracci and Op de Beeck, 2016; Watson et al., 2016).

Although lower-level image properties account for the majority of the variance in responses to intact images at early stages, there remains a significant amount of variance to be explained at later stages of processing. For example, although category-specific patterns of response to intact images persisted well beyond the duration of the stimulus, patterns of response to scrambled images were evident only when the stimulus was present. The persistence of these neural responses to intact images suggests an important role for recurrent processing of the image, which is likely to be driven by top-down semantic representations (Lamme and Roelfsema, 2000; DiCarlo and Cox, 2007; Kriegeskorte et al., 2008; Naselaris et al., 2009; Connolly et al., 2012; Mur and Kriegeskorte, 2014). Indeed, Clarke et al. (2015) showed that accuracy in categorization using MEG data was enhanced by combining visual and semantic models.

It is also possible that differences in the patterns of response between intact and scrambled images reflect sensitivity to image properties that are disrupted by either scrambling process. An important property of natural images is that they contain strong statistical dependencies, such as location-specific combinations of orientation and spatial frequency corresponding to image features such as edges (Marr and Hildreth, 1980). Indeed, the character and extent of these statistical dependencies is likely to be diagnostic for different classes of images (O’Toole et al., 2005; Rice et al., 2014). The scrambling procedure disrupts many of the statistical relationships between the elements. So, it is possible that image manipulations that can preserve these higher-level visual properties (Freeman and Simoncelli, 2011) might generate responses that are more similar to the intact images. Indeed, it is possible that neural representations underlying higher-level visual properties and the corresponding semantic properties that they convey may be the same.

In conclusion, we have found that distinct category-specific patterns of neural response emerge at ∼80 ms after stimulus onset and can persist for at least 800 ms. Using scrambled images, we show that early stages of these category-specific patterns can be explained by lower-level image properties. However, the differences in the neural responses to intact and scrambled images at later stages of processing also reveal the importance of higher-level semantic properties.

## Synthesis

The decision was a result of the Reviewing Editor Tatyana Sharpee and the peer reviewers coming together and discussing their recommendations until a consensus was reached. A fact-based synthesis statement explaining their decision and outlining what is needed to prepare a revision is listed below.

Previous comments have been adequately addressed.
